# BMScope: A scoping review to chart the evolving clinical study landscape in brain and leptomeningeal metastasis

**DOI:** 10.1093/neuonc/noae140

**Published:** 2024-08-02

**Authors:** Vinton W T Cheng, Richard Heywood, Rasheed Zakaria, Rebecca Burger, Kieran Zucker, Siddarth Kannan, Muhammad Alifian Remifta Putra, Amanda Fitzpatrick, Gary Doherty, Paul Sanghera, Michael D Jenkinson, Carlo Palmieri

**Affiliations:** Leeds Institute of Medical Research, St James’s University Hospital, University of Leeds, Leeds, UK; Department of Cancer and Genomic Sciences, University of Birmingham, Birmingham, UK; Department of Oncology, St James’s University Hospital, Leeds Teaching Hospitals NHS Trust, Leeds, UK; Department of Molecular and Clinical Cancer Medicine, University of Liverpool, Liverpool, UK; Imperial College Healthcare NHS Trust, London, UK; Department of Oncology, St James’s University Hospital, Leeds Teaching Hospitals NHS Trust, Leeds, UK; Leeds Institute of Data Analytics, University of Leeds, Leeds, UK; Department of Oncology, St James’s University Hospital, Leeds Teaching Hospitals NHS Trust, Leeds, UK; School of Medicine, University of Central Lancashire, Preston, UK; Department of Internal Medicine, Cipto Mangunkusumo Hospital, Jakarta, Indonesia; Comprehensive Cancer Centre, King’s College London, London, UK; Department of Medical Oncology, Guy’s and St Thomas’ NHS Foundation Trust, London, UK; Cambridge University Hospitals NHS Foundation Trust, Cambridge, UK; Department of Oncology, Queen Elizabeth Hospital Birmingham, University Hospitals Birmingham NHS Foundation Trust, Birmingham, UK; Department of Clinical and Molecular Pharmacology, University of Liverpool, Liverpool, UK; Institute of Systems, Molecular and Integrative Biology, University of Liverpool, Liverpool, UK

**Keywords:** brain metastasis, intracranial endpoints, leptomeningeal metastasis, scoping review

## Abstract

**Background:**

Recent studies have challenged the notion that patients with brain metastasis (BM) or leptomeningeal metastasis (LM) should be excluded from systemic therapy clinical trials. This scoping study summarizes the BM/LM clinical studies published between 2010 and 2023.

**Methods:**

MEDLINE, CINAHL, CAB Abstracts, PsycINFO, Cochrane Library, HINARI, International Pharmaceutical Abstracts, PubMed, Scopus, Web of Science, and EMBASE electronic databases were searched on June 21, 2021. An updated search was performed on February 21, 2023. Eligible studies investigated a therapeutic intervention in solid tumor patients with BM and/or LM and reported a patient outcome. Extracted study-level data, including study type, publication date, geographical location, number of BM/LM patients in the study, primary tumor type, and type of therapeutic intervention, were collected.

**Results:**

4921 unique studies were eligible for analysis. The key finding is that BM/LM clinical research is expanding globally, both in observational studies and clinical trials. Despite the shift over time toward a higher proportion of systemic therapy trials, the majority still do not include patients with symptomatic disease and lack reporting of BM/LM-specific endpoints. Globally, there has been a trend to more international collaboration in BM/LM clinical studies.

**Conclusions:**

Our analysis of the BM/LM literature charts the evolving landscape of studies involving this previously excluded population. Given the increasing clinical research activity, particularly involving late-stage systemic therapy trials, it is imperative that due consideration is given to the intracranial activity of new investigational agents. Wider adoption of standardized reporting of intracranial-specific endpoints will facilitate the evaluation of relative intracranial efficacy.

**Key Points**

- Growing involvement of brain metastasis (BM) patients in systemic drug clinical trials.
- Clinical trials focused on leptomeningeal disease, BM-targeting multimodality therapy and supportive care are lacking.
- Intracranial-specific endpoints must be prioritized.

**Importance of the Study**

Parenchymal brain metastasis (BM) and leptomeningeal metastasis (LM) are increasingly observed in cancer patients. Traditionally, patients with BM/LM were excluded from participation in systemic therapy trials. Several recently published clinical trials have demonstrated the efficacy of systemic therapies against BM. It is unclear whether these recent trials remain rare events, or represent a genuine rise in BM/LM clinical research activity.

We show that there has been a growing number of published BM/LM clinical studies since 2010 along with greater international collaboration and inclusion of BM/LM patients in systemic therapy trials. The reporting of intracranial efficacy endpoints in clinical trials remains variable.

With expanding interest in BM/LM management, it is imperative there is a coordinated approach in the design and reporting of clinical trials. This scoping study highlights the changing trends in BM/LM clinical research and potential areas for improvement. This will inform various stakeholders including policymakers, pharmaceutical companies, and clinical triallists.

Intracranial metastatic disease is chiefly characterized by parenchymal brain metastasis (BM) and leptomeningeal metastasis (LM). BM occurs through the hematogenous dissemination of cancer cells from the primary tumor, which extravasate across the blood-brain barrier (BBB) and colonize the brain parenchyma. LM also occurs through metastatic seeding from the circulation into the cerebrospinal fluid (CSF)-filled space and onto the leptomeninges, either through the arachnoid–CSF or the blood–CSF barriers.^1^ Though a relatively uncommon site of metastasis, both BM and LM remain a major cause of mortality and morbidity in affected patients,^2^ having profound implications on a patient’s social, psychological, and physical well-being.^3,4^ Certain cancers and cancer subtypes are more associated with BM and LM, particularly lung cancer, malignant melanoma, and human epidermal growth factor receptor-2 (HER2)-positive or triple-negative breast cancers.^5,6^ The prevalence of BM and LM in solid tumor types is rising, in part due to improved survival of cancer patients, as both primary and metastatic disease in extracranial sites are better controlled, and due to higher rates of detection from more widespread use of diagnostic neuroimaging.^7,8^

Despite the increasing prevalence of BM and LM in oncological practice, the management of affected patients remains challenging. Local therapies, such as stereotactic radiosurgery (SRS) and image-guided surgery, which have facilitated substantial improvements in patient outcomes, remain the treatment of choice with systemic therapies more commonly used as salvage.^9–11^ However, the development of systemic therapy for BM/LM has been slow, hampered by the limited therapeutic efficacy of systemically delivered agents due to impaired penetration across the blood–brain tumor and the blood–CSF barriers.^12^ Therefore, therapeutic strategies to overcome or bypass these barriers, such as through intrathecal administration for LM, are required to enable adequate accumulation of drug concentrations within the brain parenchyma or the CSF space to cause anti-tumor cytotoxicity. The relative dearth of proven systemic options is further compounded by the historical practice of systematically excluding BM and LM patients from clinical trials testing systemic anticancer therapies.^13^

The lack of treatment options and the poor survival of patients with BM and LM have contributed to a sense of therapeutic nihilism. Several recent large phase 2/3 clinical trials have demonstrated the clinical efficacy of a range of systemic agents in treating BM.^14–19^ These studies highlight the potential for new therapeutic options, which has led to a renewed focus on BM/LM research, with several international consensus guidelines published on how to overcome the expected challenges in clinical studies including BM/LM patients in the future.^20,21^

Recognizing that we may be reaching an inflection point in the clinical management of BM/LM, this scoping review charts clinical studies published from January 2010 to February 2023, which investigated the management of either BM, LM, or both. Using a narrative approach, our aim was to gain insight into the changing trends in BM/LM clinical research from the perspective of time, geography, primary tumor site, and clinical interventions. The scope of this review excludes articles solely focused on primary brain tumors and hematological malignancies.

## Methods

### Search Strategy and Literature Search

We conducted a systematic search of the published literature on June 28, 2021 and reported our findings according to the extension to the Preferred Reporting Items for Systematic Reviews and Meta-analyses statement (PRISMA-S). The following electronic databases were searched: MEDLINE (Ovid), CINAHL (EBSCOhost), CAB Abstracts (Ovid), PsycINFO (Ovid), Cochrane Library (Cochrane), Health InterNetwork Access to Research Initiative (Hinari Research for Health), International Pharmaceutical Abstracts (EBSCOhost), PubMed (NCBI), Scopus (Elsevier), Web of Science (Clarivate Analytics), and EMBASE (Ovid). An updated search was performed on February 21, 2023. Additional search was performed in electronic databases for conference abstracts from the European Association of Neuro-Oncology (EANO), Society of Neuro-Oncology (SNO), European Society of Medical Oncology (ESMO), and American Society of Clinical Oncology (ASCO), published from 2015 to 2023. A final trawl of studies identified from the US Food & Drug Administration (FDA) Oncology (Cancer)/Hematologic Malignancies Approval Notifications and the WHO Globus Index Medicus was performed. The full search strategy and number of studies found from each database are shown in Supplementary Tables 1 and 2.

### Study Selection

All titles and abstracts were independently screened by at least 2 study authors (V.W.T.C., R.H., R.B., R.Z., A.F., S.K., and M.A.R.P.) after initial de-duplication. The inclusion criteria were any clinical study, published in English as a full-text article involving patients with cancer and BM and/or LM. Additionally, the study must describe a therapeutic intervention and patient-relevant outcome(s). A “therapeutic intervention” was defined as any form of action used to remediate a health problem, which included pharmacological, surgical, radiotherapeutic and psychological measures. The therapeutic intervention did not need to be targeted directly at the intracranial metastasis. A “patient-relevant outcome” was defined as any reported outcomes or endpoints in the study anticipated to have a direct impact on the patient’s health or experience related to BM and/or LM, which could include survival, symptom control, and quality of life.

Excluded studies included: those with fewer than 5 BM/LM patients; articles where the full text could not be accessed and where the relevant information could not be extracted from the abstract; articles published in a language other than English; studies in patients with primary brain tumors or hematological malignancies only; and animal studies. Where studies with overlapping datasets were present, the article with the largest and most up-to-date cohorts were included. Nested studies from large clinical trials were included. Conflicts were resolved by consensus. All authors reviewed the included studies for relevance/completeness of search and highlighted any missing studies (<1% of total included studies).

### Data Extraction

A custom-designed electronic data extraction tool was piloted and refined. Six authors (V.W.T.C., R.H., R.B., R.Z., S.K., and M.A.R.P.) independently undertook data extraction for the following: first author, last author, study acronym (if applicable), study type, date of publication, country of data collection, number of BM/LM patients in the study, primary tumor type, type of therapeutic intervention investigated, and imaging or clinical biomarkers (if applicable). Since the aim of this study was to summarize all published articles relating to BM/LM, study quality was not assessed. Clinical studies were categorized as either clinical trials, defined as research studies that prospectively assign human participants to 1 or more health-related interventions to evaluate the effect on health outcomes,^22^ or observational studies.

### Data Analysis and Visualization

Collated data were transformed and mapped to standardized terms listed in Supplementary Table 3. Data processing and data visualization were performed in R (v4.3.2, R project, The R Foundation). Descriptive statistics were used to summarize the data, relating to the trend of publications over time, proportion of BM/LM studies, number of tumor sites studied, distribution of studies per tumor site, geographical distribution of studies, linkage between countries according to study collaboration, relationships between number of publications, country-specific population size and cancer prevalence, distribution of study interventions and trend of study interventions over time. Graphical representation of the data employed area graphs, pie charts, pyramid plots, UpSet plots, geographical maps, network plots, bubble charts, and stacked column charts.

Statistical analysis involved linear regression to examine the relationship between a number of published observational studies/clinical trials or a number of published BM/LM studies (dependent variables) and time (independent variable). Regression analysis was performed to compare differences in slope between 2 or more trends. A chi-squared (*χ*^2^) test or a Fisher's exact test was used to measure associations between the distribution of BM/LM trial phases, BM/LM patient profiles in phases 3 trials and trialed therapeutic modalities. All statistical analyses were 2-sided, reported at a significance level of .05 and performed in GraphPad Prism (v.10.2.3; GraphPad Software).

## Results

In total 48 222 studies were identified from the electronic search, 14 723 duplicate entries were removed and 30 207 were excluded as they related to nonhuman subjects, did not test a therapeutic intervention, included less than 5 BM/LM patients, related solely to primary brain malignancy or hematological malignancies, and/or did not report a patient-relevant outcome. After the assessment of the full text for eligibility, 2967 studies were included for analysis. An additional 123 study abstracts were deemed eligible for inclusion in the analysis. The rapid update conducted in February 2023 identified a further 1629 eligible records, giving a combined total of 4921 unique studies qualitatively synthesized for this review. A summary of the study selection process is shown in Figure S1.

### Total Academic Output of Clinical Studies Including BM/LM Patients Has Been Increasing

**Figure 1.**
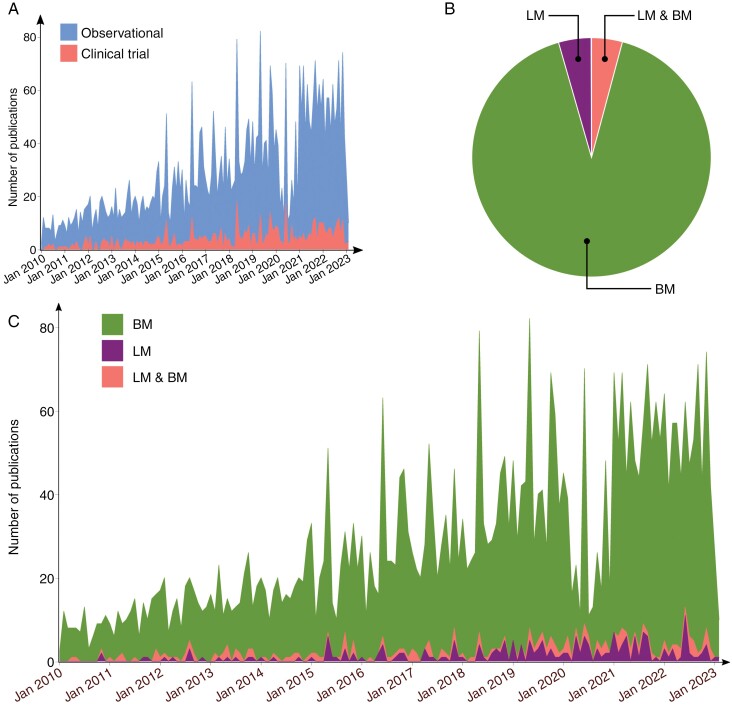
(A) Graph of the number of publications relating to BM/LM over time, separated by observational study vs. clinical trial. (B) Proportions of studies reporting on BM alone, LM alone or both and (C) area graph showing time trend of published studies according to BM alone, LM alone, or both.

Based on the temporal distribution of publications, there was an increasing clinical investigation of BM/LM from 2010 to 2023 (Figure 1A). Of the 627 clinical trials, 79 studies were published between 2010 and 2013, growing to 232 and 316 studies between 2014 and 2018, and 2019 and 2023, respectively, with a significant, albeit modest, increase in published trials over that period (regression coefficient, *β* = 0.5151; *P* < .0001). Similarly, observational studies saw a significant rise in publication rate, with 518, 1423, and 1939 studies published over the same time periods, respectively (*β* = 3.086; *P* < .0001), which was significantly faster growth than the published trials (regression analysis; *P* < .0001). Although a rising trend in publications was observed over the last 13 years, there was a noticeable drop in studies published after the last quarter (Q4) of 2019. Highlighting the granular change, in the first quarter (Q1) of 2010, there was 1 published clinical trial, by 2019 Q4 this peaked at 31, but 1 year later (2020 Q4) saw a marked reduction to only 7 published clinical trials. For nonclinical studies, the number of studies rose from 21 in 2010 Q1 to 136 in 2019 Q4, before falling to 15 in 2020 Q4. Since then, however, there has been a return in trend to an increasing number of BM/LM studies.

Out of all the included published studies, the majority involved patients with parenchymal BM (91.30%). The remaining proportion of studies was evenly split between patients with LM alone (4.10%) and both BM/LM (4.61%) (Figure 1B). The temporal trend in a number of studies published was not consistent across all metastasis groups. For BM, the number of studies published rose from 597 between 2010 and 2013, to 1595 between 2014 and 2018 (+167.2% rise compared to 2010–2012), then rising to 2126 between 2019 and 2023 (+33.3% rise compared to 2014–2018). Meanwhile, LM studies started from a much smaller baseline of 51 studies published between 2010 and 2013, but then rose steadily from 2014–2018 to 2019–2023, with 117 (+129.4% rise compared to 2010–2012) and 220 (+88.0% rise compared to 2014–2018) studies, respectively. The overall trend showed a significant rise in published BM studies (*β* = 3.244; *P* < .0001) and a significant, though less obvious, increase in published studies investigating interventions for LM (*β* = 0.2398; *P* < .0001) or both BM and LM together (*β* = 0.1172; *P* < .0001). These findings highlight the disparity in the publication rate of BM versus LM studies (regression analysis; *P* < .0001).

### Primary Tumor Sites Prioritized in BM/LM Studies Are Consistent With Known Disease Prevalence

**Figure 2.**
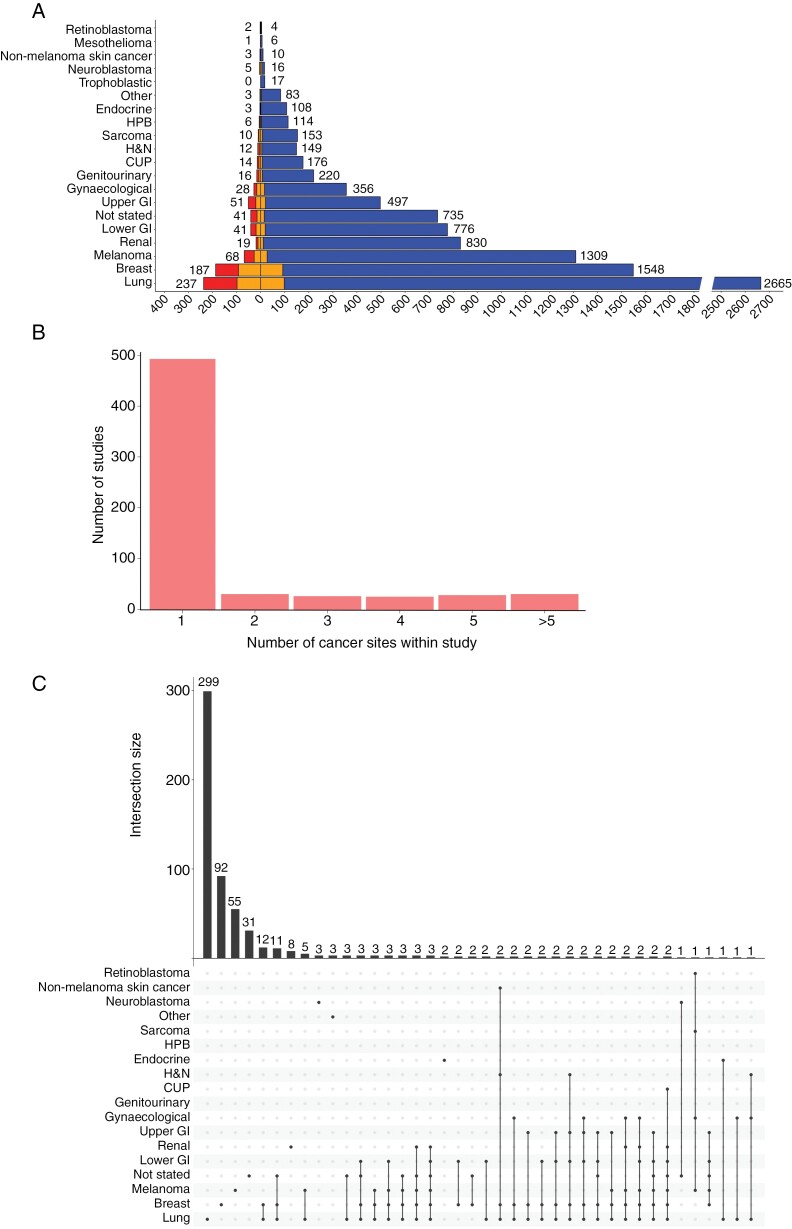
(A) Pyramid plot of all published BM/LM studies separated by cancer site and whether the study included BM-only patients (right bar), LM-only patients (left bar), or both (middle bars). (B) Number of published clinical trials reporting on BM patients with different primary cancer sites. (C) UpSet plot illustrating a number of publications according to the cancer sites included in clinical trials. Other: germ cell, neuroendocrine, and thymus.

The frequency of tumor sites investigated by the published BM/LM studies matched the disease prevalence. The top 5 tumor sites studied were lung (*n* = 2812), breast (*n* = 1646), melanoma (*n* = 1367), renal (*n* = 839), lower GI (*n* = 799) and upper GI (*n* = 531) (Figure 2A). Likewise, LM studies focused on lung (*n* = 239), breast (*n* = 188), melanoma (*n* = 69), lower GI (*n* = 41), upper GI (*n* = 52), and gynecological malignancies (*n* = 28). Clinical trials including BM/LM patients mostly focused on single tumor sites (494 out of 628 studies). Over one-sixth of clinical trials studied 3 or more different tumor sites (105 out of 628 studies) (Figure 2B). Except for 1 study, BM/LM clinical trials encompassing 2 or more tumor sites always included 1 of the top 3 tumor sites: lung, breast or melanoma (Figure 2C). Lung cancer patients were usually included in multiple tumor site studies, corresponding to the high prevalence of BM/LM in this cohort.^23^

### Demographic and Intervention Shift in Clinical Trials Between 2010 and 2023

**Figure 3.**
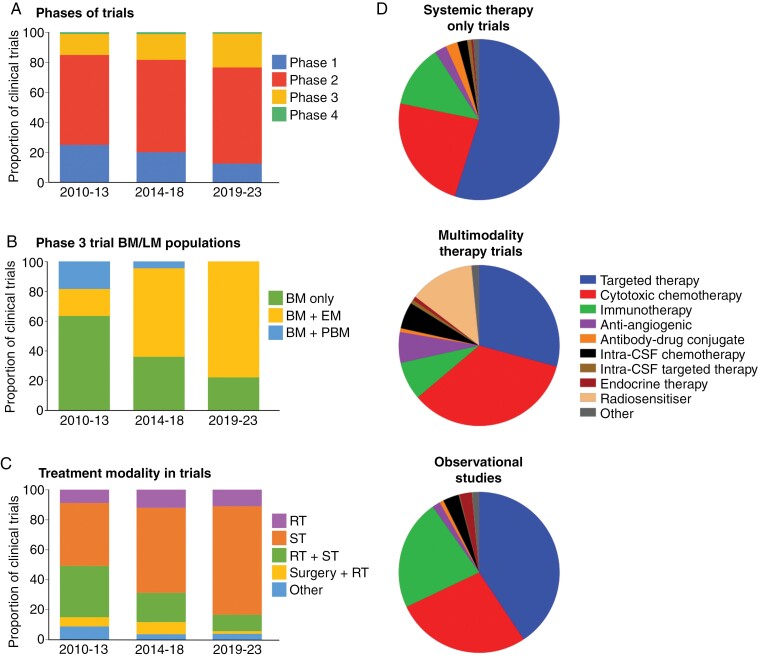
(A) Distribution of clinical trials over time according to the phase of the study: phase 1, phase 2, phase 3, or phase 4. (B) Distribution of phase 3 clinical trials over time enrolling patients with BM and extracranial metastases (BM + EM), BM and primary brain malignancy (BM + PBM), or BM only. (C) Distribution of published trials over time according to treatment modality/modalities—radiotherapy (RT), systemic therapy (ST), both radiotherapy and systemic therapy (RT + ST), both surgery and radiotherapy (surgery + RT), and other—under investigation. (D) A multitude of systemic therapy interventions have been investigated in studies including BM/LM patients, across trials testing solely systemic therapies, multimodality interventions and in observational studies. Other interventions include cell-based therapies, intra-cerebrospinal fluid (CSF) immunotherapy, localized chemotherapy, nanoparticle therapy, and stem cell transplant.

From 2010 to 2013, 25.3% (20/79) of clinical trials were phase 1 trials, with 13.9% (11/79) phase 3 trials. By the latter part of the decade (2019–2023) phase 1 trials comprised 13.0% (41/316), whilst phase 3 trials accounted for 22.2% (70/316) of all published clinical trials. This reveals a trend towards more later-phase clinical trials conducted over time, although this change was not statistically significant (*χ*^2^ test; *P* = .0723; Figure 3A). Focusing specifically on phase 3 trials, we observed a sequential increase in the number of published studies over the past decade (11 during the period 2010–2013, 39 during 2014–2018, and 70 during 2019–2023). Additionally, a changing pattern of the study populations was observed, with the majority of phase 3 studies in the early part of the decade focusing on patients with BM/LM only (or in combination with primary brain tumors), whereas by the end of the decade, most trials included BM/LM patients along with patients with extracranial metastatic disease (Fisher’s exact test; *P* = .0002; Figure 3B).

When we examined the study interventions in clinical trials across all phases, trials investigating the efficacy of systemic therapies accounted for the highest proportion of studies (33/79 (41.8%) during 2010–2013, 130/232 (56.0%) during 2014–2018, 227/316 (71.8%) during 2019–2023). This was followed by studies investigating concurrent radiotherapy and systemic therapy, and then those studying radiotherapy only. Over time there was a significant change in the distribution of therapeutic modalities tested (*χ*^2^ test; *P* < .0001) (Figure 3B). The increase in a number of phase 3 trials was almost exclusively driven by interventional systemic therapy trials (2 from 2010 to 2013, 21 from 2014 to 18, 58 from 2019 to 2023). Of note, only 6 clinical trials over this period investigated the application of supportive care interventions only, which is consistent with the well-documented lack of clinical trial evidence in palliative care as a whole.

**Figure 4.**
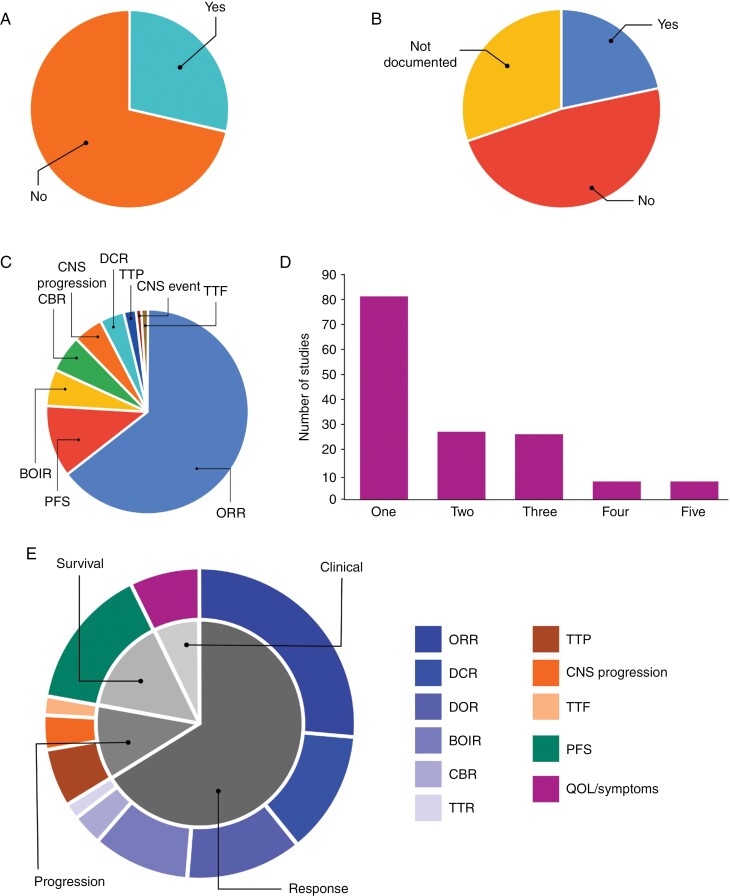
Pie charts illustrating the proportion of systemic therapy trials with (A) specified enrollment of patients with untreated or treated but progressive disease and (B) inclusion criteria permitting enrollment of BM/LM patients with symptomatic disease or requiring corticosteroids for control of symptoms. (C) The majority of systemic therapy studies, enrolling untreated or progressive BM/LM patients did not include an intracranial-specific primary endpoint, with those studies that did using a variety of different endpoints. (D) The number of intracranial-specific non-primary endpoints varied between studies, with most choosing only 1. (E) For intracranial-specific non-primary endpoints, most were focused on the radiological or clinical response, with smaller proportions measuring endpoints related to progression, survival or quality of life (QOL)/symptoms. Abbreviations: best overall intracranial response (BOIR), clinical benefit rate (CBR), central nervous system (CNS) progression, disease control rate (DCR), duration of response (DOR), objective response rate (ORR), progression-free survival (PFS), time to treatment failure (TTF), time to progression (TTP), and time to response (TTR).

A variety of systemic therapies have been subject to investigation between 2010 and 2023. Targeted agents, such as small molecule inhibitors and monoclonal antibodies, were most studied as an intervention in clinical trials (49.6%), followed by cytotoxic chemotherapy (25.7%), immunotherapy (11.6%), antiangiogenic drugs (3.2%) and antibody–drug conjugates (2.1%). For trials that only studied systemic therapies, targeted therapy comprised a greater proportion of tested interventions compared to cytotoxic chemotherapy (54.9% vs. 23.4%) (Figure 4D). In contrast, trials investigating multimodality treatments were more likely to incorporate cytotoxic chemotherapy (34.6%) rather than targeted therapy (29.2%). Moreover, radiosensitizers, for example, histone deacetylase inhibitors,^24,25^ poly-ADP ribose polymerase inhibitors,^26^ and redox modulators^27,28^ were the third largest group of systemic therapy in multimodality studies (13.1%). Meanwhile, observational studies followed a similar distribution as the clinical trials, with targeted therapy, cytotoxic chemotherapy, and immunotherapy contributing the largest fraction of systemic interventions. However, a notable difference was the greater proportion of immunotherapy observational studies including BM/LM patients compared to the systemic therapy-only trials (22.3% vs. 12.6%).

### Intracranial Outcomes Are Not Commonly Reported in BM/LM Systemic Therapy Trials

Since systemic therapy trials will usually include patients with extracranial-only disease and patients with both intracranial and extracranial disease, it is necessary that intracranial specific endpoints are reported to determine the therapeutic efficacy against BM/LM. As shown in Figure 4A, less than one-third of systemic therapy trials examined specifically required patients, either in the entire study population or as a predefined study cohort, with BM/LM disease who were untreated or had treated but progressive disease in the eligibility criteria (28.6% vs. 71.4% without specified criterion for untreated or progressive status). Even when BM/LM patients were included in these trials, just over 1 in 5 (21.5%) allowed patients with symptomatic disease or who required steroids for symptom control. In most studies, either patients were required to have asymptomatic disease (48.3%) or the symptom status was not documented (30.2%) (Figure 4B).

Intracranial-specific outcomes were mostly not reported as a primary endpoint in studies (29.2% reported vs. 70.8% not reported). In the studies that did report an intracranial-specific primary endpoint, the intracranial objective response rate (ORR) was the most popular choice (64.4%), followed by intracranial progression-free survival (11.5%) (Figure 4C). For the non-primary endpoints, intracranial outcomes were more commonly included (43.4% reported vs. 56.6% not reported) and in most cases focused on a single intracranial-specific outcome, particularly ORR (Figure 4D). The range of non-primary endpoints specifying intracranial outcomes was broad, covering the domains of response, progression, survival, and clinical assessment (Figure 4E).

### Greater International Participation in Clinical Studies Including BM/LM Patients

**Figure 5.**
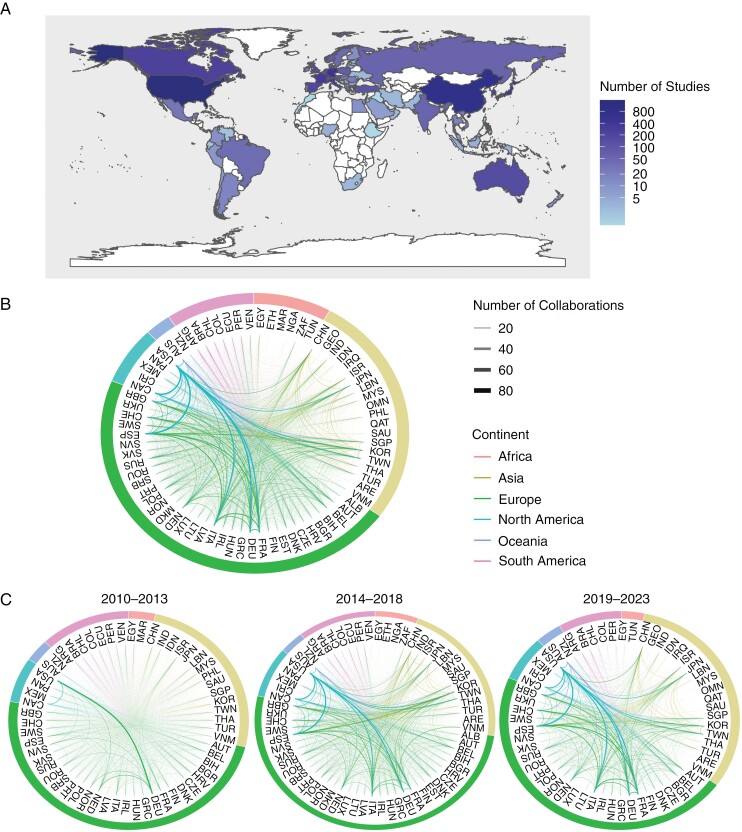
(A) Global distribution of published studies in BM/LM by country (white = no publications). Network plots, (B) combined or (C) divided by early (2010–2013), middle (2014–2018), and late (2019–2023) periods, showing changing bilateral connections in coproduced publications with the color of the line indicating participating continents and the line thickness indicating the number of collaborations between the respective nations. Country names are expressed as ISO 3166 alpha 3 country codes (see Table S5).

The geographical distribution of BM/LM studies was particularly concentrated in North America, Europe, and East Asia. Between 2010 and 2023, the United States of America accounted for 1546 published studies, which was double the output by the next nearest country, China (*n* = 751). Other nations that have contributed significantly to BM/LM research over this period were Germany (*n* = 472), Japan (*n* = 411), France (*n* = 352), Italy (*n* = 321), South Korea (*n* = 279), Canada (*n* = 250), Spain (*n* = 185), and Australia (*n* = 157) (Figure 5A). Notably, there have been collaborations involving countries from all 6 continents, with a number of low- and middle-income countries contributing to BM/LM research (Figure 5B).

Over the course of the 12 years, there has been a significant increase in international publications in BM/LM research. These copublications were strongly centered on Europe and North America. Between 2010 and 2013 (Figure 5C), the greatest frequency of copublications occurred between Germany and the United States (22), which produced one-third more copublications compared to the next highest pairings (United States and Canada, 15), and more than 4 times as many as the 10th highest combination (Netherlands and Belgium, 5). Between 2014 and 2018, copublications between United States and Canada (50) and United States and Germany (50) were joint highest, but the difference in copublications between this combination and the 3rd (United States and South Korea, 38) and 10th highest (Spain and Germany, 27) were less. Moreover, several countries from the Asia-Pacific region had entered the top 10 (South Korea, Australia, and Taiwan). This pattern continued in the latter part of the decade (2019–2023), with a similar number of copublications between the highest (United States and Germany, 65), second highest (United States and Italy, 64) and 10th highest (France and Germany, 46) combinations, and greater involvement of Asian countries (Figure 5C). Across the whole study period, most high-frequency combinations included the United States. Out of the international collaborations not involving the United States, the most productive was between Italy and Spain (62), which was the fifth most frequent combination.

### Changing Trends in Global BM/LM Research Output

**Figure 6.**
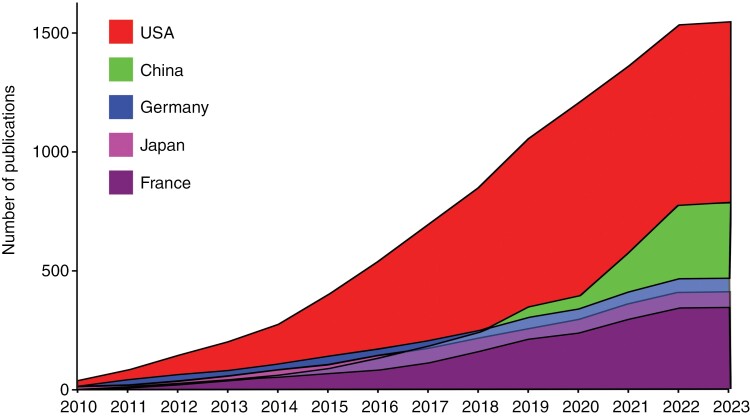
Area graph illustrating cumulative annual publications from January 2010 to February 2023 for the top 5 countries—United States of America, China, Germany, Japan, and France—with the greatest overall output for BM/LM studies.

In relation to individual output by country, the top 5 countries publishing BM/LM research were the United States, France, Germany, Japan, and China (Figure 6). The United States has consistently published the most studies per year since 2010, accounting for 42.7% (41 out of 96) of the total output and contributing to 7 of the 10 published clinical trials in 2010. Strikingly, there was a consistent drop in BM/LM research output by all 5 countries in 2020, which was followed by a recovery a year later and then stabilization for the United States, whilst France, Germany, and Japan saw a slight contraction in output in 2022. By contrast, China had only published 1 observational study in 2010, ranking the lowest of the top 5 countries at that time. However, by 2015 it had overtaken France, Germany, and Japan in the number of BM/LM publications, particularly from 2017 to 2019. Although, similar to global trends, there was a significant dip in output in 2021 China’s output then dramatically rebounded in 2021 to overtake the United States in publishing the greatest number of papers and then sustained this in 2022.

## Discussion

### Global Trends

We present a comprehensive scoping review of the BM and LM clinical research landscape between 2010 and 2023. Using a systematic approach, we identified almost 5000 clinical studies into BM or LM. This work highlights that over the course of the past decade—barring a period during the initial stages of the global pandemic caused by the severe acute respiratory syndrome-related coronavirus strain 2 (SARS-CoV-2), referred to as the Coronavirus disease (COVID-19) pandemic—there has been a consistent increase in the number of studies, both in observational and interventional studies. The anomaly in the trend occurring between 2020 and 2021, resulting from the COVID-19 pandemic, was consistent with the reported global suppression of non-COVID-19 clinical trial activity at that time, across a range of indications including cancer.^29–31^

Geographically, published BM studies were predominantly led by the United States, followed by Europe, and Asia-Pacific, with a notable increase from the latter region over time. The highest output has come from developed countries, as defined by the United Nations,^32^ but our data show that developing countries have also made important contributions to BM/LM research, with increasing international collaboration. As the global demographics transition to rising economic and social development, and with an aging population, it is anticipated that the associated increase in cancer incidence will lead to more countries participating in BM/LM research. On reviewing individual country data from the highest contributors, the research output broadly correlated with the incidence of brain metastases from different sites. There were some variations, with Japan contributing slightly less to lung BM research relative to prevalence and Australia contributing less to melanoma BM research. Of note, despite having a large cancer research footprint, the United Kingdom does not feature amongst the highest contributors to BM/LM research.

Despite a small increase in the number of BM studies investigating different treatment combinations, for example, concurrent radiotherapy with systemic treatment,^33–35^ or radiotherapy with surgery,^36–38^ the numbers remain low compared to systemic therapies alone. We have also identified that there have been very few trials investigating supportive care interventions for BM/LM patients. The QUARTZ study was a notable example of a multinational, randomized phase 3 trial, which found that in patients with non-small cell lung cancer brain metastases that were unresectable and not suitable for SRS, there was no survival or quality of life advantage gained from whole brain radiotherapy over optimal supportive care.^39^ Given that BM/LM typically develop in the terminal phase of a cancer patient’s journey, insights gained from supportive care studies such as the QUARTZ trial have the potential to positively transform patient care and, therefore, this remains an important area of unmet need.

### Inclusion Criteria

Consistent with the expected BM prevalence, most clinical trials involved lung cancer, breast cancer, or melanoma BM patients. Recently, several large trials for systemic treatments in these tumor subtypes have included patients with untreated BM and reported on their outcomes in subgroup analyses.^16,17,40–43^ Moreover, systemic therapy trials actively recruiting BM patients have reported long-term outcomes.^15,44–47^ Consequently, there is ample evidence that BM patients with lung cancer, breast cancer, or melanoma may be feasibly recruited to clinical trials. For other tumor types, the evidence base is much weaker, as they are largely incorporated with other tumor types leading to a heterogeneous population with small numbers of patients per tumor type. Therefore, the generalizability of these findings may be more challenging and has presented a barrier against the advancement of systemic treatments with proven clinical efficacy against BM from the less common tumor types, which represents an area of unmet need.

The design of systemic therapy trials that include BM patients has largely focused on patients with stable or asymptomatic disease. Although these terms have been widely used, there remains a lack of consensus regarding what classifies as asymptomatic versus symptomatic. This problematic distinction has been recently exemplified by the TRICOTEL study, a multi-center, single-arm phase 2 study involving the treatment of patients with untreated BM from melanoma, with or without a *BRAF*^V600^ mutation, who received a combination of immunotherapy (atezolizumab) and BRAF/MEK inhibitors (vemurafenib and cobimetinib). The trial was noteworthy for its inclusion of patients with symptomatic disease. However, a backlash from the cancer community over the potential misclassification of symptomatic BM led to the retraction of the published article. Subsequent re-examination of the data and analysis applied to a stricter definition of “symptomatic BM” did enable republication of the study with revised outcomes and conclusions.^48,49^ The focus on asymptomatic disease will also require a paradigm shift in the application of neuroimaging to facilitate earlier diagnosis, with careful consideration of the impact on healthcare resources and the psychosocial impact on the patient.

Over the past decade, an increasing number of clinical trials that include BM/LM patients have been published, in addition to a rise in the number of observational studies. Of these published studies, phase 3 trials accounted for a greater proportion of the increase in comparison to phase 1 trials. There has also been a large increase in the number of phase 3 trials that have BM patients alongside patients with only extracranial metastatic disease. Although a rise in the number of clinical trials is demonstrated, it remains unclear whether this correlates with an expansion in clinical trials recruiting BM patients as a proportion of total clinical trial activity. Increasing enrollment of BM/LM patients in clinical trials is dependent on taking a more pragmatic approach towards patient recruitment. In 2 recent studies of brain metastasis trial eligibility criteria, both Patel et al. (2020) and Xiao et al. (2024) found that the proportion of studies with absolute exclusion criteria of BM patients was falling coupled with an increase in studies with conditional exclusion of BM patients; thus, concluding that more nuanced inclusion of patients with CNS metastasis was increasingly being adopted.^13,50^ The loosening of inclusion criteria to permit BM patients to be recruited to clinical trials investigating novel therapeutics is a vital step, particularly given the historic underrepresentation of this patient group.

Acknowledging the widespread discriminatory practice of systematic exclusion of BM patients from clinical trials and its negative impact on the external validity of study results, the FDA has issued specific recommendations regarding best practice in study design that incorporates BM patients.^51^ Importantly, the FDA also addressed the variation in approach to BM patient recruitment, with regards to differentiating between treated/stable BM and active BM, the latter group being particularly underrepresented in clinical trials. Furthermore, there is a growing expert consensus that widening trial eligibility to include BM/LM patients is deemed a priority for future clinical trials, with recommendations from the Friends of Cancer Research highlighting the special considerations for this population that will inform the trial design.^52^

For LM patients, the progress observed in BM recruitment has been far less evident, with the numbers of clinical trials addressing this patient group sadly still lacking. A prime example of the unique challenges facing the design of prospective interventional studies in LM is the randomized phase 3 DEPOSEIN study, which demonstrated clinical benefit from the addition of intrathecal liposomal cytarabine to systemic treatment.^53^ LM studies are hindered by a slow recruitment rate, with the DEPOSEIN trial randomizing 73 patients over a 6.5 year period although this was within the projected accrual period. Moreover, the variable presentation poses a challenge to patient selection, which has been ameliorated by the establishment of standardized diagnostic criteria and clinicopathologic subtypes through the European Association of Neuro-Oncology (EANO)-European Society of Medical Oncology (ESMO) Clinical Practice Guidelines.^54^ The phase 1 BLOOM trial, investigating osimertinib in an LM-specific population with epidermal growth factor (EGFR) mutated NSCLC,^45^ demonstrated that these patients may be recruited to a well-designed early phase study, with more than 40 patients enrolled within 2.5 years. The BLOOM study also showed that an orally administered EGFR-tyrosine kinase inhibitor can be used to successfully treat LM. Maintaining the trajectory of increasing BM/LM patient engagement with clinical trials will require a coordinated push from major stakeholders in research prioritization, including policymakers, funding bodies, pharmaceutical companies and the academic community. Key strategies for achieving this goal will include stronger advocacy and by learning lessons from other underrepresented groups in cancer trials, such as rare tumor types and cancers affecting teenagers and young adults.^55,56^

The widening participation of BM patients in clinical trials represents a major paradigm shift in trial study design to investigate novel systemic therapies. Advances in drug development, especially in the fields of small molecule inhibitors and nanoparticle drug delivery systems, mean that rational design of drugs with adequate BBB penetrability is now more feasible. Moreover, the growing academic interest in BM, coupled with increasingly sophisticated preclinical models, is leading to more in vitro and in vivo pharmacological testing of drug therapies. Whilst some new therapeutics may inherently have better intracranial activity than others, it is also clear that demonstrating therapeutic effect in BM/LM may offer a competitive advantage for pharmaceutical companies, following regulatory approval and marketing authorization. Besides the larger patient pool in which the medicine may be applied, there are direct incentives for industry, for example through fast-track designation and accelerated approval programmes.^51^ Tucatinib serves as an exemplar of how medicines can benefit from the accelerated approval process following the HER2CLIMB study, which showed, in combination with capecitabine and trastuzumab, improved survival of adult patients with HER2-positive locally advanced or metastatic breast cancer. The standout feature of the HER2CLIMB study was the inclusion of a substantial proportion of patients with active or stable BM, comprising 47.5% of the total trial population.^57^ Tucatinib received Orphan Drug designation in 2017 by the FDA for combination use in pretreated HER-positive metastatic breast cancer patients with BMs, with the protected indication granted exclusivity until 17 April 2027.^58^

### Outcome Measures

The rising incidence of BM/LM, with improved detection and longer survival of cancer patients, means that there is a greater necessity for these patients to be included in clinical trials. For systemic therapy trials, the challenge arises in determining the relative efficacy in the intracranial and extracranial compartments, particularly due to the obstacle posed by the BBB to drug delivery to the CNS. Intracranial-specific endpoints provide the clearest indication for efficacy against BM/LM; however, we have highlighted the inconsistent reporting of these endpoints and their frequent omission, similar to findings in a breast cancer-specific review.^59^

There is a growing push to standardize intracranial study endpoints to facilitate improved reporting and to encourage examination of treatment efficacy in BM/LM patients.^60–63^ The relative lack of trials examining the efficacy of systemic therapies in patients with symptomatic CNS disease does negatively impact on the generalizability of trial data to the real world, since most BM/LM patients will present with symptoms. Given the risk of rapid deterioration and generally poorer outcomes associated with symptomatic BM/LM disease, testing of systemic therapies in this setting may be seen as hazardous. However, it is apparent that these clinical trials are feasible with appropriate safeguards.^44,64,65^ The Response Assessment in Neuro-Oncology (RANO) Brain Metastases working group has published a set of guiding principles in the clinical trial design of systemic therapies to evaluate the potential benefit in BM populations. In particular, the working group advocated the inclusion of BM patients earlier in the clinical development of a systemic agent and more widespread use of baseline CNS imaging. Moreover, it proposed specific design considerations based on 3 levels of anticipated CNS activity, with corresponding endpoints.^66^ Based on these recommendations, the assessment of intracranial activity in new investigational drugs should be considered according to the recruited patient population (Table S4).

Similarly, the RANO-LM working group have produced a consensus document to promote the standardization of response assessments in LM patients. The guidance hinges on 3 key elements: neurological examination, CSF analysis for tumor cells and neuraxis imaging.^67^ Subsequent evaluation of the MRI assessment tool, however, highlighted the ongoing challenges to consistently apply radiological response assessment in LM, with a notable lack of acceptable inter-rater concordance in the reporting according to the Leptomeningeal Assessment in Neuro-Oncology (LANO) scorecard.^61^ Thus, further iterative evaluation and refinement of the response criteria will be necessary to generate standardized criteria that may be applied in clinical practice or trials.

### Limitations

Despite our best attempts to undertake an extensive review of all published works related to BM and LM and to minimize bias, there are several limitations to this work. First, we cannot rule out the possibility that relevant studies may have been missed. In particular, the decision to only include studies published in English will have excluded studies published solely in other languages. To mitigate the risk of missing data, we utilized a wide range of sources to gather the data encompassing not only full-text databases, but also conference abstracts and the FDA approval list. Since the scope of this review was to describe the research landscape, we have not attempted to assess the quality of studies included in the analysis. By reviewing only the published literature, we appreciate that the data will be skewed by publication bias, leading to an underestimation of the true scale of the research being undertaken in this field. The reported dataset does not assess the number of publications reporting early closure of BM/LM trials. This information would provide insight into the recruitment challenges that are likely still facing these patient populations despite the growing prevalence of available trials, particularly in the context of a rising incidence of BM/LM.

### Future Directions

This review represents the first attempt to exhaustively map the existing literature relating to BM/LM research and chart its progress over more than 10 years. It is apparent that we are reaching a transition in the approach to the clinical management of BM. An increasing number of studies, including late-phase clinical trials, are now including BM patients and this finding is consistent with another study that has shown the growth in BM literature, as well as the recent evolution in topics towards targeted systemic therapies.^68^ This represents a major stepwise progression in the clinical community’s attitude towards BM, which had historically viewed this condition with extreme pessimism. One hopes that soon a similar trend will be seen with LM, a disease that continues to have a dismal prognosis. Technological advancements, such as novel antibody-drug conjugates, nanopharmaceuticals and liquid biopsies using circulating tumor DNA (ctDNA) offer a huge amount of promise for future treatment options in BM/LM. Increasingly, systemic therapies are being designed to improve penetration of the BBB, and there may be a role in the future for adjunctive technologies to facilitate drug delivery across the BBB via the systemic circulation.

We demonstrate that international collaborations in BM/LM research are increasing; highlighting its growing importance. As we move towards more globalization of BM/LM clinical research, it will be necessary that trials are localized to sites with high prevalence of disease to enhance equity of trial access and to overcome recruitment issues. To do so will require a greater understanding of the global BM/LM prevalence, with more robust diagnostic pathways and national reporting systems needed. There remains a relative lack of BM/LM studies investigating multimodality therapies and supportive care; these studies will be essential in improving outcomes for BM/LM patients across a wider spectrum of disease states, from a potentially curative situation to the terminal phase of illness. This resource will allow individual researchers, funders and policymakers to identify knowledge gaps to plan the next therapeutic advances in BM/LM management.

## Supplementary material

Supplementary material is available online at *Neuro-Oncology* (https://academic.oup.com/neuro-oncology).

noae140_suppl_Supplementary_Material

## References

[1] Freret ME , Boire A. The anatomic basis of leptomeningeal metastasis. J Exp Med. 2024;221(4):e20212121. 38451255 10.1084/jem.20212121 PMC10919154

[2] Cagney DN , Martin AM, Catalano PJ, et al Incidence and prognosis of patients with brain metastases at diagnosis of systemic malignancy: a population-based study. Neuro Oncol. 2017;19(11):1511–1521. 28444227 10.1093/neuonc/nox077 PMC5737512

[3] Verhaak E , Gehring K, Hanssens PEJ, Sitskoorn MM. Health-related quality of life of patients with brain metastases selected for stereotactic radiosurgery. J Neurooncol. 2019;143(3):537–546. 31073966 10.1007/s11060-019-03186-z PMC6591192

[4] Wu A , Colon GR, Lim M. Quality of life and role of palliative and supportive care for patients with brain metastases and caregivers: a review. Front Neurol. 2022;13:806344. 35250815 10.3389/fneur.2022.806344 PMC8893046

[5] Kuksis M , Gao Y, Tran W, et al The incidence of brain metastases among patients with metastatic breast cancer: a systematic review and meta-analysis. Neuro Oncol. 2021;23(6):894–904. 33367836 10.1093/neuonc/noaa285 PMC8168821

[6] Nayak L , Lee EQ, Wen PY. Epidemiology of brain metastases. Curr Oncol Rep. 2012;14(1):48–54. 22012633 10.1007/s11912-011-0203-y

[7] Berghoff AS , Schur S, Fureder LM, et al Descriptive statistical analysis of a real life cohort of 2419 patients with brain metastases of solid cancers. ESMO Open. 2016;1(2):e000024. 27843591 10.1136/esmoopen-2015-000024 PMC5070252

[8] Smedby KE , Brandt L, Backlund ML, Blomqvist P. Brain metastases admissions in Sweden between 1987 and 2006. Br J Cancer. 2009;101(11):1919–1924. 19826419 10.1038/sj.bjc.6605373 PMC2788258

[9] Park K , Bae GH, Kim WK, et al Radiotherapy for brain metastasis and long-term survival. Sci Rep. 2021;11(1):8046. 33850188 10.1038/s41598-021-87357-x PMC8044241

[10] Lippitz B , Lindquist C, Paddick I, et al Stereotactic radiosurgery in the treatment of brain metastases: the current evidence. Cancer Treat Rev. 2014;40(1):48–59. 23810288 10.1016/j.ctrv.2013.05.002

[11] Ng PR , Choi BD, Aghi MK, Nahed BV. Surgical advances in the management of brain metastases. Neurooncol Adv. 2021;3(Suppl 5):v4–v15. 34859228 10.1093/noajnl/vdab130 PMC8633760

[12] Deeken JF , Loscher W. The blood–brain barrier and cancer: transporters, treatment, and Trojan horses. Clin Cancer Res. 2007;13(6):1663–1674. 10.1158/1078-0432.CCR-06-2854 17363519

[13] Patel RR , Verma V, Miller AB, et al Exclusion of patients with brain metastases from cancer clinical trials. Neuro Oncol. 2020;22(4):577–579. 31900480 10.1093/neuonc/noz246 PMC7158639

[14] Reungwetwattana T , Nakagawa K, Cho BC, et al CNS response to osimertinib versus standard epidermal growth factor receptor tyrosine kinase inhibitors in patients with untreated EGFR-mutated advanced non-small-cell lung cancer. J Clin Oncol. 2018;36(33):3290–3297. 10.1200/JCO.2018.78.3118 30153097

[15] Tawbi HA , Forsyth PA, Algazi A, et al Combined nivolumab and ipilimumab in melanoma metastatic to the brain. N Engl J Med. 2018;379(8):722–730. 30134131 10.1056/NEJMoa1805453 PMC8011001

[16] Murthy RK , Loi S, Okines A, et al Tucatinib, trastuzumab, and capecitabine for HER2-positive metastatic breast cancer. N Engl J Med. 2020;382(7):597–609. 31825569 10.1056/NEJMoa1914609

[17] Montemurro F , Delaloge S, Barrios CH, et al Trastuzumab emtansine (T-DM1) in patients with HER2-positive metastatic breast cancer and brain metastases: exploratory final analysis of cohort 1 from KAMILLA, a single-arm phase IIIb clinical trial(☆). Ann Oncol. 2020;31(10):1350–1358. 32634611 10.1016/j.annonc.2020.06.020

[18] Bachelot T , Romieu G, Campone M, et al Lapatinib plus capecitabine in patients with previously untreated brain metastases from HER2-positive metastatic breast cancer (LANDSCAPE): a single-group phase 2 study. Lancet Oncol. 2013;14(1):64–71. 23122784 10.1016/S1470-2045(12)70432-1

[19] Freedman RA , Gelman RS, Anders CK, et al; Translational Breast Cancer Research Consortium. TBCRC 022: a phase II trial of neratinib and capecitabine for patients with human epidermal growth factor receptor 2-positive breast cancer and brain metastases. J Clin Oncol. 2019;37(13):1081–1089. 30860945 10.1200/JCO.18.01511 PMC6494354

[20] Lin NU , Wefel JS, Lee EQ, et al; Response Assessment in Neuro-Oncology (RANO) group. Challenges relating to solid tumour brain metastases in clinical trials, part 2: neurocognitive, neurological, and quality-of-life outcomes. A report from the RANO group. Lancet Oncol. 2013;14(10):e407–e416. 23993385 10.1016/S1470-2045(13)70308-5

[21] Preusser M , Winkler F, Collette L, et al Trial design on prophylaxis and treatment of brain metastases: lessons learned from the EORTC brain metastases strategic meeting 2012. Eur J Cancer. 2012;48(18):3439–3447. 22883982 10.1016/j.ejca.2012.07.002

[22] World Health Organization. *Clinical Trials*; 2020; https://www.who.int/news-room/questions-and-answers/item/clinical-trials#:~:text=What%20is%20a%20clinical%20trial,the%20effects%20on%20health%20outcomes. Date accessed February 11, 2024.

[23] Gillespie CS , Mustafa MA, Richardson GE, et al Genomic alterations and the incidence of brain metastases in advanced and metastatic NSCLC: a systematic review and meta-analysis. J Thorac Oncol. 2023;18(12):1703–1713. 37392903 10.1016/j.jtho.2023.06.017

[24] Choi CYH , Wakelee HA, Neal JW, et al Vorinostat and concurrent stereotactic radiosurgery for non-small cell lung cancer brain metastases: a phase 1 dose escalation trial. Int J Radiat Oncol Biol Phys. 2017;99(1):16–21. 28816142 10.1016/j.ijrobp.2017.04.041

[25] Deutsch E , Moyal EC, Gregorc V, et al A phase 1 dose-escalation study of the oral histone deacetylase inhibitor abexinostat in combination with standard hypofractionated radiotherapy in advanced solid tumors. Oncotarget. 2017;8(34):56199–56209. 28915584 10.18632/oncotarget.14147 PMC5593555

[26] Chabot P , Hsia TC, Ryu JS, et al Veliparib in combination with whole-brain radiation therapy for patients with brain metastases from non-small cell lung cancer: results of a randomized, global, placebo-controlled study. J Neurooncol. 2017;131(1):105–115. 27655223 10.1007/s11060-016-2275-x PMC5258788

[27] Zeng YC , Wu R, Xing R, et al Radiation-enhancing effect of sodium glycididazole in patients suffering from non-small cell lung cancer with multiple brain metastases: a randomized, placebo-controlled study. Cancer Radiother. 2016;20(3):187–192. 27052296 10.1016/j.canrad.2016.02.008

[28] Kim MM , Parmar H, Cao Y, et al Whole brain radiotherapy and RRx-001: two partial responses in radioresistant melanoma brain metastases from a phase I/II clinical trial A TITE-CRM Phase I/II Clinical Trial. Transl Oncol. 2016;9(2):108–113. 27084426 10.1016/j.tranon.2015.12.003 PMC4833892

[29] McDonald K , Seltzer E, Lu M, et al Quantifying the impact of the COVID-19 pandemic on clinical trial screening rates over time in 37 countries. Trials. 2023;24(1):254. 37013558 10.1186/s13063-023-07277-1 PMC10071259

[30] Nishiwaki S , Ando Y. COVID-19 pandemic and trends in clinical trials: a multi-region and global perspective. Front Med (Lausanne). 2021;8:812370. 35004791 10.3389/fmed.2021.812370 PMC8739772

[31] Audisio K , Lia H, Robinson NB, et al Impact of the COVID-19 pandemic on non-COVID-19 clinical trials. J Cardiovasc Dev Dis. 2022;9(1):19. 35050229 10.3390/jcdd9010019 PMC8781416

[32] DESA U. *World Economic Situation and Prospects 2022*; 2023. https://desapublications.un.org/file/728/download?_ga=2.138849843.1008521368.1674517733-1769987317.1674517733. Date accessed January 24, 2023.

[33] Chua D , Krzakowski M, Chouaid C, et al Whole-brain radiation therapy plus concomitant temozolomide for the treatment of brain metastases from non-small-cell lung cancer: a randomized, open-label phase II study. Clin Lung Cancer. 2010;11(3):176–181. 20439193 10.3816/CLC.2010.n.022

[34] Liu J , Xu J, Ye W, et al Whole-brain radiotherapy combined with anlotinib for multiple brain metastases from non-small cell lung cancer without targetable driver mutation: a single-arm, phase II study. Clin Med Insights Oncol. 2022;16:11795549221079185. 35250325 10.1177/11795549221079185 PMC8891900

[35] Palmer JD , Prasad RN, Fabian D, et al Phase I study of trametinib in combination with whole brain radiation therapy for brain metastases. Radiother Oncol. 2022;170:21–26. 35367525 10.1016/j.radonc.2022.03.016

[36] Mahajan A , Ahmed S, McAleer MF, et al Post-operative stereotactic radiosurgery versus observation for completely resected brain metastases: a single-centre, randomised, controlled, phase 3 trial. Lancet Oncol. 2017;18(8):1040–1048. 28687375 10.1016/S1470-2045(17)30414-X PMC5560102

[37] Kocher M , Soffietti R, Abacioglu U, et al Adjuvant whole-brain radiotherapy versus observation after radiosurgery or surgical resection of one to three cerebral metastases: results of the EORTC 22952-26001 study. J Clin Oncol. 2011;29(2):134–141. 21041710 10.1200/JCO.2010.30.1655 PMC3058272

[38] Brown PD , Ballman KV, Cerhan JH, et al Postoperative stereotactic radiosurgery compared with whole brain radiotherapy for resected metastatic brain disease (NCCTG N107C/CEC·3): a multicentre, randomised, controlled, phase 3 trial. Lancet Oncol. 2017;18(8):1049–1060. 28687377 10.1016/S1470-2045(17)30441-2 PMC5568757

[39] Mulvenna P , Nankivell M, Barton R, et al Dexamethasone and supportive care with or without whole brain radiotherapy in treating patients with non-small cell lung cancer with brain metastases unsuitable for resection or stereotactic radiotherapy (QUARTZ): results from a phase 3, non-inferiority, randomised trial. Lancet. 2016;388(10055):2004–2014. 27604504 10.1016/S0140-6736(16)30825-X PMC5082599

[40] Peters S , Camidge DR, Shaw AT, et al; ALEX Trial Investigators. Alectinib versus crizotinib in untreated ALK-positive non-small-cell lung Cancer. N Engl J Med. 2017;377(9):829–838. 28586279 10.1056/NEJMoa1704795

[41] Soria JC , Ohe Y, Vansteenkiste J, et al; FLAURA Investigators. Osimertinib in untreated EGFR-mutated advanced non-small-cell lung cancer. N Engl J Med. 2018;378(2):113–125. 29151359 10.1056/NEJMoa1713137

[42] Wolf J , Seto T, Han JY, et al; GEOMETRY mono-1 Investigators. Capmatinib in MET exon 14-mutated or MET-amplified non-small-cell lung cancer. N Engl J Med. 2020;383(10):944–957. 32877583 10.1056/NEJMoa2002787

[43] Shaw AT , Bauer TM, de Marinis F, et al; CROWN Trial Investigators. First-line lorlatinib or crizotinib in advanced ALK-positive lung cancer. N Engl J Med. 2020;383(21):2018–2029. 33207094 10.1056/NEJMoa2027187

[44] Davies MA , Saiag P, Robert C, et al Dabrafenib plus trametinib in patients with BRAF(V600)-mutant melanoma brain metastases (COMBI-MB): a multicentre, multicohort, open-label, phase 2 trial. Lancet Oncol. 2017;18(7):863–873. 28592387 10.1016/S1470-2045(17)30429-1 PMC5991615

[45] Yang JCH , Kim SW, Kim DW, et al Osimertinib in patients with epidermal growth factor receptor mutation-positive non-small-cell lung cancer and leptomeningeal metastases: the BLOOM study. J Clin Oncol. 2020;38(6):538–547. 31809241 10.1200/JCO.19.00457 PMC7030895

[46] Bartsch R , Berghoff AS, Furtner J, et al Trastuzumab deruxtecan in HER2-positive breast cancer with brain metastases: a single-arm, phase 2 trial. Nat Med. 2022;28(9):1840–1847. 35941372 10.1038/s41591-022-01935-8 PMC9499862

[47] Tripathy D , Tolaney SM, Seidman AD, et al ATTAIN: phase III study of etirinotecan pegol versus treatment of physician’s choice in patients with metastatic breast cancer and brain metastases. Future Oncol. 2019;15(19):2211–2225. 31074641 10.2217/fon-2019-0180 PMC7466911

[48] Dummer R , Tawbi H. Retraction and republication-TRICOTEL: defining symptomatic brain metastases in clinical trials. Lancet Oncol. 2023;24(8):e327. 37459871 10.1016/S1470-2045(23)00292-9

[49] Dummer R , Queirolo P, Gerard Duhard P, et al Atezolizumab, vemurafenib, and cobimetinib in patients with melanoma with CNS metastases (TRICOTEL): a multicentre, open-label, single-arm, phase 2 study. Lancet Oncol. 2023;24(12):e461–e471. 37459873 10.1016/S1470-2045(23)00334-0

[50] Xiao H , Vaidya R, Hershman DL, Unger JM. Impact of broadening trial eligibility criteria on the inclusion of patients with brain metastases in cancer clinical trials: time series analyses for 2012–2022. J Clin Oncol. 2024;42(16):1953–1960. 10.1200/JCO.23.01777 38537158

[51] FDA US. Cancer Clinical Trial Eligibility Criteria: Brain Metastases. Guidance for Industry; 2020. https://www.fda.gov/media/121317/download. Date accessed January 23, 2023.

[52] Lin NU , Prowell T, Tan AR, et al Modernizing clinical trial eligibility criteria: recommendations of the American Society of Clinical Oncology-Friends of Cancer Research Brain Metastases Working Group. J Clin Oncol. 2017;35(33):3760–3773. 28968165 10.1200/JCO.2017.74.0761

[53] Le Rhun E , Wallet J, Mailliez A, et al Intrathecal liposomal cytarabine plus systemic therapy versus systemic chemotherapy alone for newly diagnosed leptomeningeal metastasis from breast cancer. Neuro Oncol. 2020;22(4):524–538. 31637444 10.1093/neuonc/noz201 PMC7158648

[54] Le Rhun E , Weller M, van den Bent M, et al; EANO Guidelines Committee and ESMO Guidelines Committee. Electronic address: clinicalguidelines@esmo.org. Leptomeningeal metastasis from solid tumours: EANO–ESMO clinical practice guideline for diagnosis, treatment and follow-up. ESMO Open. 2023;8(5):101624. 10.1016/j.esmoop.2023.101624 PMC10619142 37863528

[55] Gaspar N , Fern L. Increasing access to clinical trials and innovative therapy for teenagers and young adults with cancer—a multiple stakeholders and multiple steps process. Prog Tumor Res. 2016;43:38–49. 10.1159/000447043 27595355

[56] Panageas KS. Clinical trial design for rare cancers: why a less conventional route may be required. Expert Rev Clin Pharmacol. 2015;8(6):661–663. 26517109 10.1586/17512433.2015.1088382 PMC4724195

[57] Lin NU , Murthy RK, Abramson V, et al Tucatinib vs placebo, both in combination with trastuzumab and capecitabine, for previously treated ERBB2 (HER2)-positive metastatic breast cancer in patients with brain metastases: updated exploratory analysis of the HER2CLIMB Randomized Clinical Trial. JAMA Oncol. 2023;9(2):197–205. 36454580 10.1001/jamaoncol.2022.5610 PMC9716438

[58] Shah M , Wedam S, Cheng J, et al FDA approval summary: tucatinib for the treatment of patients with advanced or metastatic HER2-positive breast cancer. Clin Cancer Res. 2021;27(5):1220–1226. 33055172 10.1158/1078-0432.CCR-20-2701

[59] Bhogal T , Cameron D, Palmieri C. Central nervous system disease in phase III studies for advanced HER2 positive breast cancer: a review. Breast. 2022;63:85–100. 35344688 10.1016/j.breast.2022.03.013 PMC8961215

[60] Lin NU , Lee EQ, Aoyama H, et al; Response Assessment in Neuro-Oncology (RANO) group. Response assessment criteria for brain metastases: proposal from the RANO group. Lancet Oncol. 2015;16(6):e270–e278. 26065612 10.1016/S1470-2045(15)70057-4

[61] Le Rhun E , Devos P, Boulanger T, et al; European Organisation for Research and Treatment of Cancer (EORTC) Brain Tumor Group (BTG) Central Nervous System (CNS) Metastases Committee and the EORTC BTG Imaging Committee. The RANO leptomeningeal metastasis group proposal to assess response to treatment: lack of feasibility and clinical utility and a revised proposal. Neuro Oncol. 2019;21(5):648–658. 30715514 10.1093/neuonc/noz024 PMC6502503

[62] Chukwueke UN , Wen PY. Use of the response assessment in neuro-oncology (RANO) criteria in clinical trials and clinical practice. CNS Oncol. 2019;8(1):CNS28. 30806082 10.2217/cns-2018-0007 PMC6499019

[63] Aizer AA , Lamba N, Ahluwalia MS, et al Brain metastases: a Society for Neuro-Oncology (SNO) consensus review on current management and future directions. Neuro Oncol. 2022;24(10):1613–1646. 35762249 10.1093/neuonc/noac118 PMC9527527

[64] Yang H , Deng Q, Qiu Y, et al Erlotinib intercalating pemetrexed/cisplatin versus erlotinib alone in Chinese patients with brain metastases from lung adenocarcinoma: a prospective, non-randomised, concurrent controlled trial (NCT01578668). ESMO Open. 2017;2(Suppl 1):e000112. 29147576 10.1136/esmoopen-2016-000112 PMC5682358

[65] Dagogo-Jack I , Oxnard GR, Evangelist M, et al Phase II Study of Lorlatinib in patients with anaplastic lymphoma kinase-positive lung cancer and CNS-specific relapse. JCO Precis Oncol. 2022;6:e2100522. 35584349 10.1200/PO.21.00522 PMC9848561

[66] Camidge DR , Lee EQ, Lin NU, et al Clinical trial design for systemic agents in patients with brain metastases from solid tumours: a guideline by the Response Assessment in Neuro-Oncology Brain Metastases Working Group. Lancet Oncol. 2018;19(1):e20–e32. 29304358 10.1016/S1470-2045(17)30693-9

[67] Chamberlain M , Junck L, Brandsma D, et al Leptomeningeal metastases: a RANO proposal for response criteria. Neuro Oncol. 2017;19(4):484–492. 28039364 10.1093/neuonc/now183 PMC5464328

[68] Chen J , Williams M, Huang Y, Si S. Identifying topics and evolutionary trends of literature on brain metastases using latent dirichlet allocation. Front Mol Biosci. 2022;9:858577. 35720132 10.3389/fmolb.2022.858577 PMC9201447

