# Supplementary material for: BMScope: A scoping review to chart the evolving clinical study landscape in brain and leptomeningeal metastasis
Source: Neuro Oncol. 2024 Aug 2;26(12):2193–207. doi: 10.1093/neuonc/noae140 (PMC11630544; doi:10.1093/neuonc/noae140)
Supplement: noae140_suppl_Supplementary_Material [file noae140_suppl_supplementary_material.docx]

Records identified through database searching
(n = 47563)

Additional records identified through other sources
(n = 659)

Records after duplicates removed
(n = 33499)

Records screened
(n = 33499)

Records excluded
(n = 30207)

Full-text articles assessed for eligibility
(n = 3292)

Full-text articles excluded, with reasons
(n = 325)

Studies included in qualitative synthesis
(n = 4921)

Additional records identified in rapid update
(n = 1629)

**Fig S1.** PRISMA flow diagram mapping out the number of records identified, included and excluded.

| **Intervention** |
| --- |
| (Intervention OR Ablat* OR Service OR Agent OR Intravenous OR Medic* OR Immunotherap* OR Inhibit* OR Radiotherap* OR Radiation OR Stereotactic OR Radiosurgery OR IMRT OR VMAT OR Conform* OR Surg* OR Drug OR Therap* OR Test OR Biomarker OR Diagnos* OR Predict* OR Prognos* OR Chemotherap* OR “Small molecule” OR “Monoclonal antibody” OR Tablet OR Supportive OR Physiotherap* OR Drain OR Psycholog* OR Talking OR CBT OR Cognit*) |
| **Clinical studies** |
| (“Clinical stud*” OR Population OR Prospective OR Trial OR Non-randomi* OR Retrospective OR Randomi* OR Retrospective OR “Human study” OR First-in-man OR Phase or Control* OR Case-control OR Cohort) |
| **Clinical outcomes** |
| (“Patient outcome*” OR Harm OR “Side effect*” OR “Hazard ratio” OR Survival OR “Relapse free” OR Overall OR Death OR “Quality of life” OR Symptom OR Paresis OR Pain OR Seizure OR Weakness OR Cognition OR PROM OR “Patient experience” OR “Patient reported outcome measure” or Recurrence OR Death OR Fatality OR Mortality OR Progression OR Response OR “Complete response” OR “Response rate” OR “Partial response” OR Relapse) |
| **Intracranial metastasis** |
| (Metasta* OR Cancer* OR Tumour OR Secondary OR Tumor or Malignan*) AND (“Brain” OR “Neuro*” OR “Grey matter” OR “Cerebellum*” OR “Supratentorial” OR “Intracranial” OR “Central nervous system” OR CNS OR Cerebral OR Infratentorial OR Brainstem OR “White matter” OR Leptomening* OR Mening* OR “Neoplastic mening*”) |

**Table S1.** Key terms adapted for search in electronic databases.

| List of sources searched: | Date of search | Total number of results found |
| --- | --- | --- |
| CAB Abstracts | 28/3/20 | 13 |
| CINAHL | 28/3/20 | 1292 |
| EBM – Cochrane Reviews | 28/3/20 | 11 |
| EMBASE | 28/3/20 | 59 |
| International Pharmaceutical Abstracts | 28/3/20 | 6 |
| Medline | 28/3/20 | 393 |
| PsycINFO | 28/3/20 | 23 |
| PubMed | 21/2/23 | 12710 |
| Scopus | 21/2/23 | 17397 |
| WoS | 21/2/23 | 30333 |
| ASCO Abstracts | 30/5/23 | 3339 |
| ESMO Abstracts | 30/5/23 | 333 |
| SNO Abstracts | 30/5/23 | 234 |
| EANO Abstracts | 30/5/23 | 38 |

**Table S2.** Number of studies identified per electronic database based on search strategy.

| **Group** | **Standardised term** |
| --- | --- |
| Tumour type | \| Breast \| \| --- \| \| CUP \| \| Endocrine \| \| Genitourinary \| \| Germ cell \| \| Germ cell tumour \| \| Gynaecological \| \| Head & neck \| \| HPB \| \| Lower GI \| \| Lung \| \| Melanoma \| \| Mesothelioma \| \| Nephroblastoma \| \| Neuroblastoma \| \| Neuroendocrine \| \| Non-melanoma skin cancer \| \| Not stated \| \| Renal \| \| Retinoblastoma \| \| Sarcoma \| \| Thymus \| \| Trophoblastic \| \| Upper GI \| |
| Intervention | \| Clinical biomarker \| \| --- \| \| Definitive treatment to other metastasis \| \| Definitive treatment to primary tumour \| \| Imaging \| \| Intensive care \| \| Multidisciplinary approach \| \| Neurosurgery \| \| Radiotherapy \| \| Stem cell transplant \| \| Supportive \| \| Supportive \| \| Systemic therapy \| |
| Neurosurgery | \| Cerebral shunt \| \| --- \| \| Computer assisted neurosurgery \| \| CSF access device \| \| Cyst drainage \| \| Fluorescence guided neurosurgery \| \| Gross total resection \| \| Image guided neurosurgery \| \| Intralesional/cavitary drugs \| \| Intraoperative adjunct \| \| Intraoperative monitoring \| \| Laser interstitial thermal therapy \| \| Minimally invasive surgery \| \| Multiple craniotomies \| \| Preoperative mapping \| \| Supramarginal resection \| \| Ventriculostomy \| |
| Radiotherapy | \| Brachytherapy \| \| --- \| \| Cavity SRS \| \| Conformal RT \| \| CSF targeted radionuclide \| \| FSRT \| \| Intracavitary radionuclide \| \| Intraoperative RT \| \| Not specified \| \| Proton therapy \| \| RT boost \| \| SRS \| \| Systemic radioisotope \| \| WBRT \| |
| Systemic therapy | \| Anti-angiogenic \| \| --- \| \| Antibody-drug conjugate \| \| Cell therapy \| \| Cytotoxic chemotherapy \| \| Endocrine therapy \| \| Immunotherapy \| \| Intra-CSF chemotherapy \| \| Intra-CSF immunotherapy \| \| Intra-CSF targeted therapy \| \| Localised chemotherapy \| \| Nanoparticle therapy \| \| Not specified \| \| Radiosensitiser \| \| Stem cell transplant \| \| Targeted therapy \| |
| Imaging | \| CSF imaging \| \| --- \| \| CT \| \| Functional brain mapping \| \| Imaging contrast \| \| MRI \| \| PET \| \| Surface guidance \| \| Ultrasound \| |
| Biomarker | \| Blood \| \| --- \| \| Circulating cells \| \| Clinical measurement \| \| Cognitive test \| \| CSF \| \| ctDNA \| \| Tumour \| |
| Supportive care | \| Anti-cerebral oedema \| \| --- \| \| Anticoagulation \| \| Anticonvulsants \| \| Cognition-enhancing medication \| \| Complementary and alternative medicines \| \| Not specified \| \| Nutritional management \| \| Palliative care \| \| Patient education \| \| Psychological therapy \| \| Radioprotection \| \| Rehabilitation \| \| Service improvement \| \| Stem cell harvest \| \| Steroids \| \| Treatment adjunct \| \| VTE treatment/prophylaxis \| |

**Table S3.** Standardised terms used to map tumour type and interventions.

| **Patient population** | **Proposed intracranial endpoints** |
| --- | --- |
| BM/LM-free | Culminative incidence rates |
| Locally treated and non-progressive BM/LM | CNS progression rates, iPFS, iTTF, iTTP |
| Asymptomatic/symptom-controlled and untreated, or treated/progressive BM/LM (non-measurable) | CNS progression rates, iPFS, iTTF, iTTP |
| Asymptomatic/symptom-controlled and untreated, or treated/progressive BM/LM (measurable) | iORR, iDCR, iDOR, BOIR, iCBR, iTTR |
| Symptomatic, untreated or treated/progressive BM/LM | iORR, iDCR, iDOR, BOIR, iCBR, iTTR, iQOL, CNS symptoms |

**Table S4**. Proposed intracranial-specific endpoints in trials to demonstrate intracranial efficacy for a systemic agent, according to recruited BM/LM population.

| **Country name** | **ISO 3166a3 code** |  | **Country name** | **ISO 3166a3 code** |
| --- | --- | --- | --- | --- |
| Albania | ALB |  | North Macedonia | MKD |
| Argentina | ARG |  | Malaysia | MYS |
| Australia | AUS |  | Mexico | MEX |
| Austria | AUT |  | Morocco | MAR |
| Belgium | BEL |  | Netherlands | NLD |
| Bosnia and Herzegovina | BIH |  | New Zealand | NZL |
| Brazil | BRA |  | Nigeria | NGA |
| Bulgaria | BGR |  | Norway | NOR |
| Canada | CAN |  | Oman | OMN |
| Chile | CHL |  | Pakistan | PAK |
| China | CHN |  | Panama | PAN |
| Colombia | COL |  | Peru | PER |
| Costa Rica | CRI |  | Philippines | PHL |
| Croatia | HRV |  | Poland | POL |
| Czechia | CZE |  | Portugal | PRT |
| Denmark | DNK |  | Qatar | QAT |
| Ecuador | ECU |  | Romania | ROU |
| Egypt | EGY |  | Russian Federation | RUS |
| El Salvador | SLV |  | Saudi Arabia | SAU |
| Estonia | EST |  | Serbia | SRB |
| Ethiopia | ETH |  | Singapore | SGP |
| Finland | FIN |  | Slovakia | SVK |
| France | FRA |  | Slovenia | SVN |
| Georgia | GEO |  | South Africa | ZAF |
| Germany | DEU |  | Spain | ESP |
| Greece | GRC |  | Sweden | SWE |
| Hong Kong | HKG |  | Switzerland | CHE |
| Hungary | HUN |  | Taiwan | TWN |
| India | IND |  | Thailand | THA |
| Indonesia | IDN |  | Tunisia | TUN |
| Iran | IRN |  | Türkiye | TUR |
| Iraq | IRQ |  | United Kingdom of Great Britain and Northern Ireland (the) | GBR |
| Ireland | IRL |  | Ukraine | UKR |
| Israel | ISR |  | United Arab Emirates | ARE |
| Italy | ITA |  | United States of America (the) | USA |
| Japan | JPN |  | Venezuela (Bolivarian Republic of) | VEN |
| Jordan | JOR |  | Viet Nam | VNM |
| Korea (the Republic of) | KOR |  |  |  |
| Latvia | LVA |  |  |  |
| Lebanon | LBN |  |  |  |
| Lithuania | LTU |  |  |  |
| Luxembourg | LUX |  |  |  |

**Table S5.** Countries mapped to corresponding International Standard for country codes (ISO 3166 alpha 3).
